# Good in Providing Oral Care, but we Could be Better—Nursing Staff Identification of Improvement Areas in Oral Care

**DOI:** 10.1177/23779608211045258

**Published:** 2021-10-01

**Authors:** Maria Andersson, Mona Persenius

**Affiliations:** Department of Health Science, Faculty of Health, Science and Technology, 4209 Karlstad University, Karlstad, Sweden

**Keywords:** nursing staff, oral care, oral health, older people, residential care, satisfaction

## Abstract

**Introduction:**

Oral care to older people in short-term care units is a complex and challenging everyday practice for nursing staff. Oral care research and knowledge about prerequisites and obstacles is extensive. However, there is a lack of knowledge about how nursing staff in short-term care units describe their satisfaction about provided oral care in order to maintain older people's oral health.

**Objective:**

The purpose of this study was to describe how nursing staff perceive their satisfaction of oral care provided for older people in short-term care units and to identify oral care improvements.

**Methods:**

This study reports on the results of two open-ended questions that were part of a larger study. Informants (*n* = 54) were nursing staff working in the involved short-term care units in municipalities from both densely and sparsely populated regions in central and northern Sweden. The answers to the open-ended questions were analyzed using content analysis.

**Results:**

The analysis yielded one main category; “*Working together to improve satisfaction with older people's oral care”* and four subcategories: “*Older people's oral health,*” “*Consideration and respect for the older person's autonomy,*” “*Having access to adequate products,*” and “*Working together in the same direction.*”

**Conclusion:**

Identification of older people's oral health problems together with adequate nursing intervention will increase older people's health outcomes and quality of life. However, regardless of work role, the nursing staff might have difficulty changing their behavior or transforming intentions into actions. Oral care is a complicated and proactive practice that requires nursing staff's attention as well as both educational and organizational initiatives. Working in a supportive and collaborative relationship provides prerequisites for optimal oral care in short-term care units.

## Introduction

Registered nurses (RNs) and other nursing staff such as enrolled nurses (ENs) and nurse assistants (NAs) have a responsibility for providing oral care to older people with care dependency (Huotari & Havrdovà, 2016; Pudlowski, 2015). Poor oral health in this population might subsequently lead to increased risk of poor general health outcomes (Badewy et al., 2021). Oral care reduces the bacterial colonization in the oral cavity (Cecona et al., 2010) prevents deterioration in oral health (Gaszynska et al., 2014; Janssens et al., 2017), and the development of aspiration-associated pneumonia (Teramoto et al., 2015). A systematic review by Khadka et al. (2021) showed that aspiration-associated pneumonia occurred less in older people who received professional oral care compared with no such care. Despite this, oral care is one of the most overlooked nursing procedures in residential care (Coker et al., 2017; Hilton et al., 2016) such as short-term care (STC) units. These units are a form of intermediate care and are concerned with a person's transition between hospital and home (Melis et al., 2004). The purpose of a STC unit is to promote recovery, prevent unnecessary hospital admissions, support hospital discharge, and enable older people to maintain their independence for as long as possible (Melis et al., 2004).

## Background

Oral care is a component of nursing care and involves person-centered approaches when assessing the oral cavity, disrupting plaque, reducing salivary microorganisms, cleansing and moisturizing tissues to prevent plaque-associated diseases, and improving psychological well-being (Coker et al., 2013). Recommendations for oral care suggest that nursing staff should assess older peoples’ oral health status as soon as possible (National Institute for Health and Care Excellence [NICE], 2016). Daily oral care involves tooth brushing with fluoride toothpaste at least twice a day, tooth brushing of full or partial dentures, and, if possible, the use of the older person’ choice of oral care products (NICE, 2016). Oral care is complex and multifaceted, and according to McCrae (2011) determined by different conditions, such as local and national policies, procedures, research evidence, professional and social norms, person-related values, and experiences.

Nursing staff perceive oral care to older people to be an important aspect of nursing care (Andersson et al., 2020; Ek et al., 2018), but it has low-priority status compared to other nursing care (Coker et al., 2017; Ek et al., 2018; Hilton et al., 2016). Oral care is often spontaneous, is often of varying quality, and it is not always based on evidence (Coker et al., 2017; Ek et al., 2018; Hilton et al., 2016), and even if documentation exists, nursing staff rarely consult the oral care plans (Coker et al., 2017). Yet, a cross-sectional study showed that nursing staff in STC units were overall satisfied with the provided oral care (Andersson et al., 2019).

## Review of Literature

Nursing staff working in STC units attend to older people with an increasing number of severe physical and cognitive limitations and who are at risk for impaired oral health, decreased health, and well-being. Oral care to older people is a complex and challenging everyday practice for nursing staff in STC units. Oral care research and knowledge about prerequisites and obstacles is extensive. However, there is a lack of knowledge about how nursing staff in STC units describe their satisfaction about provided oral care. Such knowledge might identify strengths and weaknesses in oral care that older people receive in STC units.

Purpose of this study was to describe how nursing staff perceive their satisfaction of oral care provided for older people in STC units and to identify oral care improvements.

## Methods

### Design

This study reports on the results of two open-ended questions that were part of a larger quantitative study and are reported elsewhere (Andersson et al., 2019). The study was a part of the larger research project called Swallowing Function, Oral Health and Food Intake in Old Age (SOFIA) that explored different aspects related to oral health in older people in STC units (Hägglund et al., 2017).

### Sample

Convenience sampling was used to recruit nursing staff in 23 STC units in 19 municipalities from both densely and sparsely populated regions in central and northern Sweden. All nursing staff (RNs, ENs, NAs) working part- or fulltime in the 23 STC units were invited to participate.

RNs have a university education and licensure to provide nursing care autonomously (Nursing and Midwifery Board of Australia [NMBA], 2016a; Swedish Society of Nursing, 2017). RNs working in STC units have a more consultative approach. Most nursing care internationally (Etherton-Beer et al., 2013), as well as in Sweden (National Board of Health and Welfare [NBHW], 2014), is carried out by ENs and NAs with a lower level of nursing education.

ENs work under the direct or indirect supervision of the RN as part of the nursing care team and are responsible for their own actions in providing delegated nursing care (NMBA, 2016b). ENs undertake a Diploma of Nursing (NMBA, 2016b).

NAs are unregulated nursing staff and provide nursing care related to activities of daily living to older people in residential care facilities (Hewko et al., 2015; Schnelle et al., 2016). Their education level is high school or less (Hewko et al., 2015) and they receive a minimum of training.

### Data Collection

The participants’ understanding of oral care satisfaction were collected through the following two open-ended questions: “What are you particularly satisfied with regarding oral care in your unit?” and “Do you have any suggestions for oral care improvements?” The participants’ answers were formulated in one or more sentences.

### Procedures

Research assistants gave verbal information to the nursing staff in the STC units. Each informant received the questionnaire with the two open-ended questions together with an addressed and prepaid envelope. Informants also received written information about the aim and design of the study. Nursing staff were informed verbally and in writing that their participation was voluntary and their identity would be kept confidential. Research assistants reminded the nursing staff by visiting the STC units. The data collection took place from October 2013 to January 2016.

### Statistical Analysis

The open-ended questions in the questionnaire were analyzed using inductive qualitative content analysis with a manifest approach (Graneheim & Lundman, 2004). First, the text was read through several times to obtain a comprehensive understanding of the content. Next, the meaning units were highlighted, condensed, and labelled with codes. During the abstraction, process codes were compared based on similarities and differences, grouped into four subcategories, and then sorted into one main category. The analysis was an iterative process, moving back and forth across the phases. Tentative categories were discussed by the two authors until consensus was reached. Table 1 shows an example of the analysis process.

**Table 1.** Example of the Analysis Process.

Main category: Working together to improve satisfaction with older people's oral care			
Meaning units	Condensed meaning units	Code	Category
The nursing staff is very committed and aware of the benefits of good oral health	Very committed and aware of the benefits of good oral health	Awareness of good oral health	Older people's oral health
We are quick to see if someone has problems, for example with food intake, and to find out what is wrong and then start the right treatment to manage to eat or be pain free in the mouth	Find out what is wrong	Identification of problems	
If the older person can take care of their teeth in whole or in part, it is important they are allowed to do so	Important they are allowed to take care of their teeth	Facilitating participation	Consideration and respect (for the older person's autonomy)
We help those who want help but sometimes they say no	Want to help but sometimes they say no	Respect for their will	
We do the best we can, all patient bring their own oral care items. So sometimes you wish they had brought a better toothbrush and toothpaste	We do the best we can. Wish they had brought a better toothbrush and toothpaste	Wishing for better products	(Having) access to adequate products
I think that most of my working group try to take time with oral care, we all try to ensure that the older people have the aids needed for good oral care and health	All try to ensure that the older people have the aids needed for good oral care	Doing their best to find oral care products	
Together all staff help those who need help	Together all staff help	Together to support	(Working) together in the same direction
We try to get better. We have opened our eyes to the importance of oral care. So we work better with this	We try to get better	We are improving	

### Research Ethics

Nursing staff were informed verbally and in writing that their participation was voluntary and their identity would be kept confidential.

## Results

### Sample Characteristics

Fifty-four out of the 276 participants who answered the questionnaire answered the two open-ended questions (RNs = 10, ENs = 42, NAs = 2). Their age ranged from 22 years to 65 years and they were all women. All RNs had university education, and a majority of ENs (*n* = 37) and all NAs had postsecondary school education.

### Research Question Results

The analysis of the two open-ended questions yielded one main category “*Working together to improve satisfaction with older people's oral care”* and four subcategories “*Older people's oral health,*” “*Consideration and respect for the older person's autonomy,*” “*Having access to adequate products,*” and “*Working together in the same direction.*”

### Working Together to Improve Satisfaction with Older People's Oral Care

The overall result showed that nursing staff were working in a supportive and collaborative relationship to improve satisfaction with older people's oral care.

### Older People's Oral Health

The nursing staff perceived they were good at identifying oral health problems like pain in the oral cavity and swallowing difficulties. They described it is important to assess oral health status on admission and after 6 weeks or continuously. They also described how many older people have impaired oral health combined with low physical and psychological ability. There was a perception that there are fewer older people with dental prostheses compared with previously, but the prostheses in use often no longer fit well. Therefore, nursing staff suggested the oral cavities of older persons with dental prostheses should be assessed more often. Another problem identified by nursing staff was that older people do not always want their oral cavity to be assessed. At some STC units, the nursing staff described how they were obliged to use the Revised Oral Assessment Guide (ROAG) (Andersson et al., 2002). The ROAG is included in the national quality register Senior Alert, used when admitting older persons to the STC unit and when evaluating the oral health status after 6 weeks.

Nowadays, not so many older people have dental prostheses as before, so it is important to check their mouth when they are admitted. That is what we do now thanks to Senior Alert. (EN)

At other STC units that did not use the national quality register, the nursing staff suggested that the ROAG should be a routine task carried out on all older people within 48 hr of admission to the STC unit. They also suggested oral health assessments could be performed by any of the nursing staff, and not just by RNs.

### Consideration and Respect for the Older Person's Autonomy

The nursing staff described the importance of consideration and respect regarding the older person's autonomy when providing oral care. The nursing staff see everyone as a unique person, and it is the older person's decision if they want help with oral care. The nursing staff described how they help older people with oral care on a daily basis, for example, helping brush teeth after mealtimes, however it is important that older people take care of their own oral health as much as possible.

The nursing staff are good at providing the older people with the opportunity to care for their teeth in a good way. (EN)

Nursing staff felt they are good at facilitating older people in taking care of their teeth, but it can be problematic to help older people with dementia because they do not always willingly open their mouths. However, the nursing staff could usually manage to support those who did not want to brush their teeth. They also perceived that older people themselves and their next of kin do not always find oral care to be important for many reasons, for example, worry that dental visits might be seen as too expensive. Nursing staff described how the dental professionals did not always understand how difficult it was to provide oral care to cognitively impaired older people.

Many older people do not want to participate in oral health assessments, and many become a little defensive. Although discussing this often among staff, we feel that oral health assessments sometimes violate the older people's privacy! (EN)

### Having Access to Adequate Products

According to the nursing staff, having access to adequate products is a prerequisite for implementing good oral care, and staff also considered the availability of different oral care products to be important. Usually the older people bring their own oral care products when admitted to the STC unit.

Most often the older people bring their own toothpaste and toothbrush from home! Great! Sometimes also an interdental brush. (NA)

The nursing staff get together and try to obtain the needed products, if not brought to the STC unit. Sometimes the nursing staff wished for better oral care products than what the older people had brought. However, the stock at the STC could vary, and the nursing staff suggested a supply of oral care products at each STC unit so that older people who did not bring their own oral care products could get what they needed. Labelled toothbrushes and denture mugs, and access to fluoride rinses were other suggestions for improvement.

### Working Together in the Same Direction

Nursing staff were aware of the importance of oral health for older people's well-being, and they are doing their very best in relation to the time available. Being able to consult someone on the team whenever oral health problems are identified (like pain in the mouth) is highly appreciated by the nursing staff. They perceived there are well-functioning collaborations between RNs and ENs as well as with the dental hygienist and dental care. However, because access to a dental hygienist varied among the STC units, the nursing staff believed the older people could benefit from dental outreach by dental hygienists or by referral to a dentist. They were all working in the same direction, but thought they could still make improvements.

In general, I think most staff are concerned about providing good oral care, but at the same time you can always try to improve. (EN)

Nursing staff perceived they were more focused nowadays on the older persons’ oral health and adherence to oral care recommendations is high. However, there was a reported need for improvements. Nursing staff with specific oral care knowledge collaborate with the older people and their next of kin to maintain the older people's oral health by giving them support and advice about oral care. In order to improve oral care, they described their own need for oral care education and training, regardless of role, on a regular basis led by nursing staff with specific oral care knowledge and skills or by dental professionals. For example, education and training regarding different oral care products and cleaning bridges, implants, and dentures as well as how to handle older people not wanting to participate in oral assessments.

## Discussion

This study aimed to describe how nursing staff perceive their satisfaction of oral care provided for older people in STC units and to identify oral care improvements. The findings indicate that nursing staff working together in teams including the older people and the dental hygienists, felt they were good at oral care but could be better.

### Older People's Oral Health

Nursing staff described how they were good at identifying older people's oral health problems. A previous study by Andersson et al. (2018) showed that 98% of older people in Swedish STC units had moderate to severe oral health problems, highlighting the need for better oral assessment. Regarding structured assessments, the nursing staff suggested that all nursing staff and not only RNs should have education and practice in carrying out oral health assessments. However, RNs and other nursing staff have indicated that the ROAG is difficult to use (Ek et al., 2018) and requires some degree of skill (NICE, 2016). There is a need for further studies regarding what assessment instrument is optimal in nursing environments where several work roles carry out the assessments.

The review by Salamone et al. (2013) showed that oral problems in acute medical units were not documented unless the oral problems were obvious. The use of assessment tools might have an impact on the documentation, and according to Nyongesa (2013), the use of the Oral Health Assessment Tool increases oral care documentation. Therefore, frequent structured assessments with valid and reliable instruments and evaluations of older people's oral health are recommended.

The nursing staff perceived older people sometimes could be defensive when their oral cavity was assessed. Attempts to provide oral care might trigger older people with dementia to try to avoid receiving any type of oral care (Ishii et al., 2012). On the other hand, older people themselves sometimes perceive that nursing staff do not always take action regarding their oral health problems (Andersson et al., 2018). Strategies to improve oral care among nursing staff have focused, for example, on knowledge, awareness, and attitudes (Weening-Verbree et al., 2013). In 2011, the Swedish Association of Local Authorities and Regions (SKR) took initiatives in patient safety, including the area of oral health. The follow-up report (SKR, 2018) showed the average cost of oral health education had increased as well as the number of oral care trained nursing staff, but there were major differences in education budgets between the regions (SKR, 2018). This indicates different priorities for oral care, something that might be the same worldwide. A recent literature review by Badewy et al. (2021) found that poor oral health among older people may lead to an increased risk of poor general health outcomes and have negative impact on health care costs.

Nursing staff in this study described older people with prostheses that no longer fit well, and according to Gaszynska et al. (2014) and Janssens et al. (2017) older people's oral health may deteriorate during residential care. Yildiz et al. (2013) found that patients in intensive care units using dental prostheses already had worsening of oral health and unhealthy oral mucus membranes on the fourth day compared with their status at admission. Older people using dental prostheses must be orally assessed on a regular basis.

### Consideration and Respect for the Older Person's Autonomy

The nursing staff stressed the importance of consideration and respect for the older people's autonomy and they facilitated the older people to participate in decisions regarding their oral care. However, not all of the older people wanted to open their mouth and participate in oral assessments. This made the nursing staff feel as if they violated the older people's privacy. It is well known that older people's preferences for involvement in decision-making might differ (Kiselev et al., 2018; Wiltjer et al., 2019) and that some older people want to use their energy in other ways than maintaining their oral health (Andersson et al., 2018; Niesten et al., 2013). Most strategies do not consistently show positive effects on older people's oral health (Albrecht et al., 2016). A combination of different strategies (Leeman et al., 2017) might generate improvements in oral care. For example, the three-component management intervention for oral care-refusal behavior developed by Jablonski et al. (2018), which has been shown to significantly increase older people's willingness to receive oral care.

### Having Access to Adequate Products

The nursing staff described the available oral care products in STC units did not always allow nursing staff to provide optimal oral care. According to Andersson et al. (2018) older people in STC units usually have access to a toothbrush and toothpaste, but not to other products such as dental sticks, interdental brushes, or dental floss. In both developing and developed countries, the burden of impaired oral health is particularly high among underprivileged and disadvantaged older people (Petersen & Ogawa, 2018). In Sweden, older people in STC units pay for their own oral care products (NBHW, 2014). The consequences might be that due to the costs for oral care products, older people do not receive adequate oral care (Hilton et al., 2016) unless they have next-of-kin supporting them. Meeting older people's oral care needs might be easier for the nursing staff if the overarching organization provided a supply of oral care products at each STC unit.

### Working Together in the Same Direction

Having support within the nursing staff as well as from the dental hygienists regarding the older people's oral health was of great importance. Nursing staff were aware of some nurses who do not prioritize oral care because of time and/or staff shortages. A systematic review by Pentecost et al. (2020) showed that time shortages due to all the necessary nursing care interventions are common reasons for nursing staff not consistently addressing patients’ or older people's fundamental care needs. On the other hand, having extra nursing staff did not mean the workload was perceived to be reduced (Pentecost et al., 2020). Nursing staff are often aware of oral care's importance (Andersson et al., 2019; Ek et al., 2018), but the present study indicates that nursing staff have difficulty in changing their behavior or in transforming intentions into actions. To do so might require a change within the nursing staff along with increased emphasis on the significance of oral care.

## Strengths and Limitations

The open-ended questions were part of a larger questionnaire that investigated different aspects of oral care in STC units. The low participation (54 out of 276 respondents) might be explained by high workloads having a negative impact on the respondents’ ability to answer the open-ended questions. If a larger proportion of RNs had participated, the results might have been different. RNs in STC units might have a more consultative approach and provide basic nursing care such as oral care to a lesser extent than ENs and NAs. However, the focus in this study was on oral care and statistical analysis of academic degree, work role, work experience and oral care is suggested for further investigation. Despite possible differences in the organization of care across STC units, all care services should deliver oral care according to older people's needs and preferences.

The qualitative results presented here were part of a larger quantitative study (Andersson et al., 2019). At the end of the questionnaire, two open-ended questions were asked. However, having first completed the questionnaire (Andersson et al., 2019) might have affected the way informants answered the open-ended questions.

Both authors were involved in the analysis process. The categories were thoroughly described, and quotations from participants substantiated the categories (Graneheim & Lundman, 2004). However, the nursing staff formulated their answers in one or more sentences, which in a sense made some of the answers already condensed.

The data collection period was long (2013–2016) because of other aspects of the SOFIA project. The prerequisites for nursing staff to provide oral care might have changed during the data collection period and this might have had an impact on the findings. A significant change of importance during the last year was the COVID-19 pandemic and oral health has according to Marchini and Ettinger (2020) not been the focus. For further research, it is important to have knowledge of the nursing staff's oral care satisfaction prior to the COVID-19 pandemic, and thus be able to compare their oral care satisfaction after the COVID-19 pandemic.

## Implications for Practice

- Not only RN, but also ENs and NAs should have education and rehearsal of carrying out oral health assessments
- Use of structured assessment tools might have impact on the documentation
- There is a need for increased emphasis on the significance of oral care by a combination of different strategies

## Conclusion

Identification of older people's oral health problems together with adequate nursing intervention will increase older people's health outcomes and quality of life. However, regardless of work role, the nursing staff might have difficulty changing their behavior or transforming intentions into actions. Oral care is a complicated and proactive practice that requires nursing staff's attention as well as both educational and organizational initiatives. Working in a supportive and collaborative relationship provides prerequisites for optimal oral care in STC units.
